# Prognosing post-treatment outcomes of head and neck cancer using structured data and machine learning: A systematic review

**DOI:** 10.1371/journal.pone.0307531

**Published:** 2024-07-24

**Authors:** Mohammad Moharrami, Parnia Azimian Zavareh, Erin Watson, Sonica Singhal, Alistair E. W. Johnson, Ali Hosni, Carlos Quinonez, Michael Glogauer

**Affiliations:** 1 Faculty of Dentistry, University of Toronto, Toronto, Canada; 2 Department of Dental Oncology, Princess Margaret Cancer Centre, Toronto, Canada; 3 Topic Group Dental Diagnostics and Digital Dentistry, ITU/WHO Focus Group AI on Health, Geneva, Switzerland; 4 Chronic Disease and Injury Prevention Department, Health Promotion, Public Health Ontario, Toronto, Canada; 5 Program in Child Health Evaluative Sciences, The Hospital for Sick Children, Toronto, Canada; 6 Radiation Oncology, Princess Margaret Cancer Center, University of Toronto, Toronto, Canada; 7 Schulich School of Medicine & Dentistry, Western University, London, Canada; 8 Department of Dentistry, Centre for Advanced Dental Research and Care, Mount Sinai Hospital, Toronto, Canada; University of Hong Kong, HONG KONG

## Abstract

**Background:**

This systematic review aimed to evaluate the performance of machine learning (ML) models in predicting post-treatment survival and disease progression outcomes, including recurrence and metastasis, in head and neck cancer (HNC) using clinicopathological structured data.

**Methods:**

A systematic search was conducted across the Medline, Scopus, Embase, Web of Science, and Google Scholar databases. The methodological characteristics and performance metrics of studies that developed and validated ML models were assessed. The risk of bias was evaluated using the Prediction model Risk Of Bias ASsessment Tool (PROBAST).

**Results:**

Out of 5,560 unique records, 34 articles were included. For survival outcome, the ML model outperformed the Cox proportional hazards model in time-to-event analyses for HNC, with a concordance index of 0.70–0.79 vs. 0.66–0.76, and for all sub-sites including oral cavity (0.73–0.89 vs. 0.69–0.77) and larynx (0.71–0.85 vs. 0.57–0.74). In binary classification analysis, the area under the receiver operating characteristics (AUROC) of ML models ranged from 0.75–0.97, with an F1-score of 0.65–0.89 for HNC; AUROC of 0.61–0.91 and F1-score of 0.58–0.86 for the oral cavity; and AUROC of 0.76–0.97 and F1-score of 0.63–0.92 for the larynx. Disease-specific survival outcomes showed higher performance than overall survival outcomes, but the performance of ML models did not differ between three- and five-year follow-up durations. For disease progression outcomes, no time-to-event metrics were reported for ML models. For binary classification of the oral cavity, the only evaluated subsite, the AUROC ranged from 0.67 to 0.97, with F1-scores between 0.53 and 0.89.

**Conclusions:**

ML models have demonstrated considerable potential in predicting post-treatment survival and disease progression, consistently outperforming traditional linear models and their derived nomograms. Future research should incorporate more comprehensive treatment features, emphasize disease progression outcomes, and establish model generalizability through external validations and the use of multicenter datasets.

## 1. Introduction

Head and neck cancer (HNC) is the seventh most common cancer globally, accounting for more than 660,000 new cases and 325,000 deaths annually. By 2030, the incidence of HNC is expected to rise by 30% compared to the 2020 rate, largely driven by increases in oropharyngeal cancer [1, 2]. Post-treatment recurrences and metastases are common occurrences that contribute to poor prognosis of HNC [3, 4]. The five-year relative survival rate of HNC has improved during the past decades from 54.1% (1975–84) to 66.8% (2005–2004) based on the Surveillance, Epidemiology, and End Results (SEER) data [5]. Despite the increase in survival rates, per capita death rates have risen over the last decade, reflecting the predominance of the increase in incidence over the survival rate [2]. Squamous cell carcinoma (SCC) is the most common type of HNC, constituting 90% of the cases and attracting considerable research aimed at enhancing diagnostic, prognostic, and therapeutic interventions. [6].

Traditional prognostication of HNC outcomes primarily relied on nomograms and linear models that took into account factors such as the primary tumor size and extent, lymph node involvement, and the presence of distant metastasis [3]. However, this approach inadequately addressed the inherent heterogeneity among HNC patients, leading to less accurate individual risk assessments. In response, recent models have incorporated a more diverse range of prognostic variables, including patient demographics, histopathological information, treatment details, comorbidities, and molecular markers [7, 8]. Concurrently, machine learning (ML) has emerged as a promising tool, leveraging its capacity for non-parametric modeling to analyze extensive and intricate datasets more flexibly. This approach allows for the accounting of non-linear relationships and interactions between predictors, offering a more nuanced understanding of the data [9, 10].

Although there has been a shift toward including unstructured data such as medical images in the prognosis of HNC outcomes, utilizing structured data such as tabulated clinicopathological features offers several advantages. Structured data is more amenable to systematic organization and analysis, whereas unstructured data requires extensive preprocessing to convert it into a format suitable for analysis [11, 12]. Recent advancements in deep learning (DL) have reduced the need for extensive preprocessing by substituting it with the requirement for large datasets for model development [13]. However, the scarcity of large, suitable datasets for training presents a challenge to this approach. Structured data typically comprises variables with established clinical relevance, leading to well-specified models with greater interpretability. This characteristic also makes structured data more consistent across different institutions and provides opportunities for external validation. Furthermore, the availability of structured data might facilitate the development of larger-scale prognostic models [14].

This review aims to evaluate the performance of ML models in predicting post-treatment disease progression (i.e., recurrence and metastasis) and survival outcomes in patients with HNC, utilizing structured data. This study distinctly concentrates on models that leverage clinicopathological data, routinely sourced from electronic health records (EHRs), which hold the potential for implementation in actual clinical settings. Additionally, this review will not be confined to the oral cavity but will also encompass other disease sites within HNC and provide an in-depth analysis of each site. The study will also provide an evaluation of current ML models, highlighting their strengths and weaknesses in light of rapid field advancements.

## 2. Materials and methods

### 2.1. Protocols

This systematic review was prepared according to the guidelines of the Preferred Reporting Items for Systematic Reviews and Meta-analyses (PRISMA) statement [15]. The focused question was addressed using the participant, intervention, comparison, outcomes, time, and setting (PICO-TS) criteria [16]. The study protocol was registered on the International Prospective Register of Systematic Reviews (PROSPERO) platform under the registration number CRD42023426148.

### 2.2. Search strategy

Five electronic databases including Medline, Scopus, Embase, Web of Science, and Google Scholar were used to identify publications that met the inclusion criteria. The search was conducted up to March 15, 2024, using the following combination of MeSH terms and keywords: (artificial intelligence OR AI OR machine learning OR deep learning OR neural network* OR supervised learning OR semisupervised learning OR unsupervised learning OR multilayer perceptron OR MLP) AND (oral OR head and neck OR HNC OR mouth OR oropharyn* OR hypopharyn* OR nasopharyn* OR laryn*) AND (carcinoma OR malignancy OR neoplasm OR cancer*). Besides the electronic databases, reference lists of the selected studies were manually screened.

### 2.3. Eligibility criteria

The following inclusion criteria were used to screen and assess the retrieved records:

1. being an original peer-reviewed article in English;
2. reporting on ML-based prognostication models for post-treatment survival and disease progression. Post-treatment survival outcomes included both disease-specific and overall survival for any follow-up duration, encompassing both disease-free survival and survival with disease. Disease progression outcomes comprised post-treatment loco-regional recurrence and metastasis.
3. reporting on models developed only using structured and clinicopathological data;
4. reporting on the performance of the ML and DL models.

Records were excluded if they:

1. used unstructured data; unstructured data refers to information that does not have a predefined data format or is not organized in a systematic manner. This type of data typically includes formats like text, images, audio, and video, which are not easily searchable or analyzable using conventional data processing techniques.
2. used structured data other than clinicopathological such as biochemical markers, molecular, and genomic data;
3. reported on prognostic outcomes other than survival and disease progression;
4. only reported on validation and not the development of models.

### 2.4. Focused PICO-TS question

What is the predictive performance of ML models, developed based on clinicopathological data, in informing the survival and disease progression of HNC patients?

Participants (P): HNC patients who received treatments.
Intervention (I): application of ML models in predicting post-treatment survival and disease progression outcomes
Comparison (C): ML models’ predictive performance compared to actual events and/or traditional linear models. Examples of traditional models include logistic and Cox proportional hazards (Cox PH) regressions.
Outcomes (O): performance metrics reported for binary classification or time-to-event outcomes including sensitivity (recall), specificity, precision, F1-score, accuracy, area under receiver operating characteristics (AUROC), concordance index (C-index), and Brier score.
Timeframe (T): The review will include both retrospective and prospective studies that report outcomes based on data collected from patients over varying follow-up periods to ensure the analysis reflects long-term prognostic performance. We will specify the follow-up durations for each included study in the review results.
Settings (S): The review will include studies conducted in various clinical settings, such as hospitals, cancer research centers, and academic institutions. The data will be primarily sourced from electronic health records (EHRs), which are routinely used in clinical practice.

### 2.5. Selection of studies

A two-stage screening (title-abstract, full text) was carried out by two authors independently (MM, PAZ). Title management was performed by a commercially available software program (Covidence systematic review software, Veritas Health Innovation, Melbourne, Australia). The duplicates were removed within and between the databases. The full texts of potential articles were retrieved and evaluated using an eligibility form. Any disagreements on the selection of studies were discussed and resolved. The reasons for excluding articles not meeting the eligibility criteria were reported.

### 2.6. Data extraction

Using a predesigned data extraction form, the following information was extracted from the papers that met the eligibility criteria: title, authors’ names, authors’ affiliations, database, year of publication population, sample size, case-to-control ratio, outcome measure, tumor site, tumor histology, clinicopathological features, ML models, dimensionality reduction, feature selection, resampling techniques, imbalance class correction, validation (i.e., internal vs. external), performance metrics, traditional model comparator, and authors’ conclusion.

### 2.7. Risk of bias

The systematic review assessed the quality of the included studies using the Prediction model Risk Of Bias ASsessment Tool (PROBAST) [17]. This tool, tailored for evaluating diagnostic and prognostic models, examines bias risk across four domains: participants, predictors, outcomes, and analysis. The evaluation employed 20 signaling questions, categorizing the risk of bias as low, unclear, or high. Applicability concerns for the initial three domains were similarly classified as low, unclear, or high. An overall low risk of bias was determined if all domains received a low rating. However, for prediction models developed without external validation, even if all domains were rated low, the risk of bias should be considered high unless the model was developed on a very large dataset and included internal validation [18]. Two authors (MM, PAZ) independently conducted the assessments, with cross-checking to ensure accuracy and consistency.

### 2.8. Data analysis

A qualitative methodology was employed to interpret and summarize the findings from the selected studies. This synthesis encompassed study characteristics, clinicopathological features, ML models, validation methods, and performance metrics. Subgroup analyses of the results were adapted based on the tumor site. Specifically, the following sites were considered separately: oral cavity, oropharynx, nasopharynx, hypopharynx, and larynx. When a specific subsite, such as the tongue, was investigated, it was categorized under a broader category, such as the oral cavity. If a study combined different sites without reporting performance for each site separately, they were categorized as HNC. The synthesis of the results was performed when there were at least two or more studies available for each site. When feasible, and if not reported, metrics such as the F1-score were derived from other metrics such as recall and precision. For binary classification tasks that did not incorporate time modeling, our report primarily focuses on AUROC as a threshold-independent metric and the F1-score as a threshold-dependent metric, provided these were reported. In cases where studies conducted time-to-event analyses, considering both time modeling and censored data, the primary performance metric employed was the C-index. In addition to discrimination metrics, calibration metrics were reported if they were assessed in a study. While all performance metrics were documented, only those derived from test sets (either internal or external) were utilized for synthesis. The discussion includes an analysis of the limitations and trends observed across the studies, providing insights into the current state of ML models used in this domain.

## 3. Result

### 3.1. Study selection and search results

**Fig 1:**
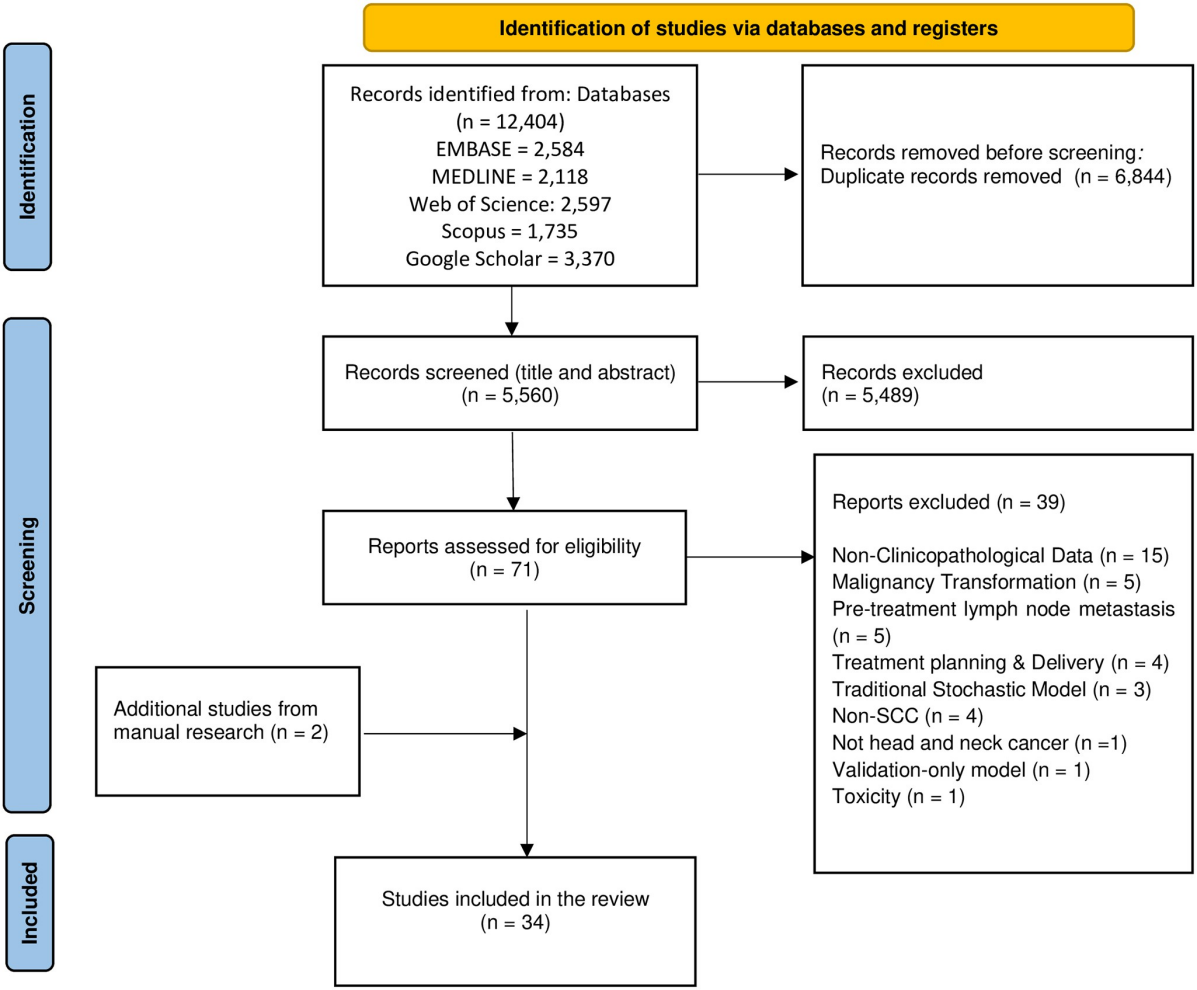
The flowchart of the search process. *From*: Page MJ, McKenzie JE, Bossuyt PM, Boutron I, Hoffmann TC, Mulrow CD, et al. The PRISMA 2020 statement: an updated guideline for reporting systematic reviews. BMJ 2021;372:n71. doi: 10.1136/bmj.n71. For more information, visit: http://www.prisma-statement.org/.

The systematic search resulted in 5,560 unique records. After screening the title and abstracts 5,489 records were excluded. From the remaining 71 records, 39 articles were excluded in the full-text reading stage: 15 articles worked on non-clinicopathological data [19–30], five articles were about preventive outcomes including early diagnosis and malignancy transformation [31–35], five articles were on pre-treatment lymph node metastasis [36–40], four articles were about the use of ML in treatment planning and delivery [41–44], three articles used traditional stochastic modeling [45–47], one article was not about HNC [48], one article validated an already available tool and did not develop a model [49], one article was about toxicity [50], and three articles were on non-SCC cancer [51–54]. After manually screening the reference lists, two other studies were added, and the final 34 included studies were processed for data extraction [55–88]. The summary of the search strategy is depicted in the PRISMA flowchart in Fig 1.

### 3.2. Assessment of methodological quality

**Fig 2:**
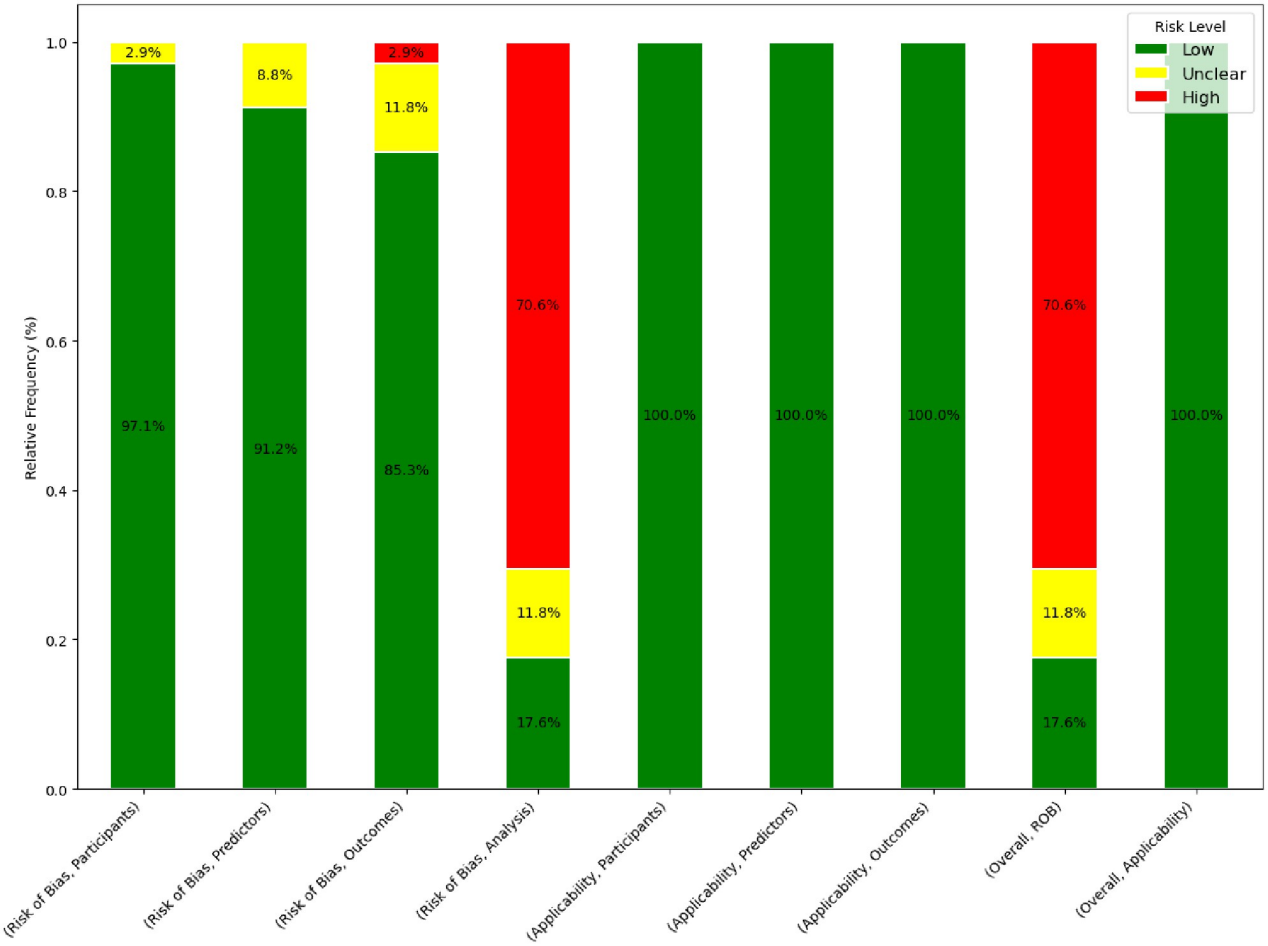
The risk of bias and applicability concerns of the included studies based on PROBAST. Abbreviations. ROB: risk of bias, PROBAST: Prediction model Risk Of Bias ASsessment Tool.

Table 1 outlines a comprehensive assessment of the risk of bias and applicability concerns. Among the 34 studies evaluated, six were assessed as exhibiting a low overall risk of bias, 24 exhibited a high risk, and four exhibited an unclear risk of bias. In the participant domain, all studies were characterized by a low risk of bias except for one with unclear risk. Concerning the predictor domain, an unclear risk of bias was identified in three studies, whereas the remaining studies were considered to have a low risk. For the outcome domain, one study was identified as high risk, four had an unclear risk, and the remainder were categorized as low risk. The most significant source of bias emerged in the analysis’s domain, with 24 studies classified as high risk. The primary contributors to this high-risk rating were small sample sizes, which resulted in a ratio of participants with the outcome to the number of predictor candidates being less than 10. Additional contributing factors included a lack of cross-validation or a test set, not accounting for complexities in data such as censoring or competing risks, the utilization of univariate analyses for feature selection, and failure to report performance metrics for both discrimination and calibration. In the analysis’s domain, four other studies exhibited an unclear risk of bias, while only six were evaluated as having a low risk. Regarding applicability concerns, all studies demonstrated a low risk across all domains (Fig 2).

**Table 1:** The risk of bias and applicability concerns of the included studies based on PROBAST.

	Author (Year)	Risk of Bias				Applicability			Overall	
		Participants	Predictors	Outcomes	Analysis	Participants	Predictors	Outcomes	ROB	Applicability
1	Tseng (2015)	+	+	?	-	+	+	+	-	+
2	Sharma (2015)	+	?	-	-	+	+	+	-	+
3	Cheng (2018)	+	?	?	-	+	+	+	-	+
4	Karadaghy (2019)	+	+	+	-	+	+	+	-	+
5	Alabi (2019)	+	?	+	-	+	+	+	-	+
6	Kim (2019)	+	+	+	-	+	+	+	-	+
7	Hung (2020)	+	+	?	?	+	+	+	?	+
8	Alabi (2020)	+	+	+	-	+	+	+	-	+
9	Alkhadar (2020)	+	+	+	-	+	+	+	-	+
10	Chu (2020)	+	+	?	-	+	+	+	-	+
11	Shan (2020)	+	+	+	-	+	+	+	-	+
12	Du (2020)	+	+	+	+	+	+	+	+	+
13	Nogay (2020)	+	+	+	-	+	+	+	-	+
14	Yu (2021)	+	+	+	+	+	+	+	+	+
15	Bourdillon (2022)	+	+	+	-	+	+	+	-	+
16	Gangli (2022)	?	+	+	-	+	+	+	-	+
17	Adeoye (2022)	+	+	+	-	+	+	+	-	+
18	Peng (2022)	+	+	+	+	+	+	+	+	+
19	Kim (2022)	+	+	+	+	+	+	+	+	+
20	Alabi (2022)	+	+	+	-	+	+	+	-	+
21	Tan (2022)	+	+	+	-	+	+	+	-	+
22	Liao (2023)	+	+	+	?	+	+	+	?	+
23	Alabi (2023)	+	+	+	-	+	+	+	-	+
24	Kotevski (2022a)	+	+	+	-	+	+	+	-	+
25	Kotevski (2023b)	+	+	+	+	+	+	+	+	+
26	Xiao (2023)	+	+	+	?	+	+	+	?	+
27	Sun (2023)	+	+	+	+	+	+	+	+	+
28	Cai (2023)	+	+	+	-	+	+	+	-	+
29	Choi (2023)	+	+	+	?	+	+	+	?	+
30	Li (2023)	+	+	+	-	+	+	+	-	+
31	Zhang (2023)	+	+	+	-	+	+	+	-	+
32	Fatapour (2023)	+	+	+	-	+	+	+	-	+
33	Alabi (2024)	+	+	+	-	+	+	+	-	+
34	Li (2024)	+	+	+	-	+	+	+	-	+

Symbols

+: low risk of bias, -: high risk of bias,?: unclear

Abbreviation

PROBAST: Prediction model Risk Of Bias ASsessment Tool

### 3.3. Study characteristics

The characteristics of the included studies are summarized in chronological order in Tables 2 and 3 for the survival and disease progression outcomes. The publication dates ranged from 2015 to 2024 with only three studies published before 2019. Out of the 34 studies analyzed, 15 were based on the US population data, with 10 of those studies being conducted by non-US institutions using SEER datasets. The sample sizes ranged from 145 to 177,714. Of the 34 studies, 24 were on survival outcomes while 11 were on disease progression; one study covered both survival and disease progression. For the survival outcome, 11 studies reported the performance of ML models for tumors located in the oral cavity, eight for the larynx, five for the oropharynx, three for the hypopharynx, and two for the nasopharynx; also six studies reported the performance of ML models for HNC without differentiating for different sites. Regarding disease progression outcomes, nine studies targeted the oral cavity, while one study focused on the larynx, and another study HNC.

**Table 2:** The characteristic of the included studies in chronological order for the survival outcome.

First Author (Year)	Authors’ Affiliation	Data Source/ Population	Sample Size	Cases Frequency N (%)	Survival Outcome Type	Tumor Site	Histological Diagnoses	Primary Treatment	Clinicopathological Features Included in Models		
									Patient-related	Treatment-related	Tumor-related
Tseng (2015)	Taiwan Republic of China	A Single Center Hospital/Southern Taiwan	Internal: 673 Train: 90% Test (10%)	426	5-year disease specific	Oral cavity	SCC	Surgery	.Age .Sex .Oral history .smoking .Alcohol .Betel-nut chewing .Family history	.Duration between onset and the beginning of treatment .CCRT (yes/no)	.Tumor site .AJCC-N .Level of metastasis in lymph node .Lymphovascular invasion .Peripheral nerve invasion .Tumor size .Clinical stage .Distant metastasis
Sharma (2015)	India	Chart review of data from ENT (Ear- Nose-Throat) and Head-Neck Department of three Tertiary Care Hospitals of Pune /India	Internal: 1025 Train: 90% Test (10%)	40.2%	NM	Oral cavity	SCC	Surgery Radiotherapy Chemotherapy	NM	.Surgery (yes/no) .Radiotherapy (yes/no) .Chemotherapy (yes/no)	.Tumor stage .1st and 5th follow up symptoms .1st and 5th follow up examination
Karadaghy (2019)	USA	National Cancer Database (NCDB)/ USA	Internal: 33,065 Train: 80% Test: 20%	16745 (50.6%)	5-year overall	Oral cavity	SCC	Surgery Radiotherapy	.Age .Sex .Race .Marital status .Type of insurance .Facility site . Household income .Urban or rural residence .Charlson-Deyo comorbidity score	. Surgical margin .No. of days from surgery to discharge .Days from diagnosis to start of radiotherapy	.Tumor site .Grading .Extracapsular spread .Perineural invasion .TNM staging
Kim (2019)	South Korea	Single Center Hospital/South Korea	Internal: 255 Train: 70% Test: 30%	44 (17.3%)	Disease-specific	Oral cavity	SCC	Surgery	.Age .Sex	.Presence of tumor at resection margin .Adjunctive radiotherapy .Adjunctive chemotherapy	.Tumor Site .Histologic Grading .TNM staging .T-stage .N-stage .Bone marrow invasion .Perineural invasion .Lymphovascular permeation .Extranodal extension .Recurrence
Hung (2020)	USA	National Cancer Institute (NCI) Surveillance, Epidemiology, and End Results (SEER)/ USA	Internal: 177,714 Train: 75% Test: 25%	N/A (continuous)	Duration of Survival	HNC	SCC	Surgery	.Age .Sex .Race .Marital status	.Type of surgical procedure	.Year and month of diagnoses .Tumor site .Tumor size .Tumor extension .Lymph nodes .AJCC staging
Alkhadar (2020)	UK	NM/Scotland	Internal: 416 Train: 75% Test: 25%	101 (24%)	5-year recurrence-free	Oral Cavity	SCC	Surgery	.Age .Sex .Pain symptoms	.Surgery choice (with or without neck dissection)	.Tumor Site .Grade .Lymphovascular invasion .Extracapsular extension .Perineural invasion .Bone invasion
Du (2020)	Australia	National Cancer Institute (NCI) Surveillance, Epidemiology, and End Results (SEER)/ USA	Internal: 3-year: 21,154 5-year: 21000 Train/Test ratio: 8:2 7:3 5:5 3:7	5-year: 60.1% 3-year: 65.0%	3-year disease-specific 5-year disease-specific	HNC	SCC	Surgery	.Age .Sex .Race .Marital status	.Surgery (yes/no)	.Grade .T-stage .N-stage .M-stage .AJCC stage .Tumor size .Tumor site
Nogay (2020)	Turkey	Cancer Imaging Achieve (TCIA)/ Canada	Internal: 300 Train: 70% Validation: 15% Test:15%	56 (19%)	Overall	HNC	SCC	Radiotherapy	.Age .Sex .HPV	.Time to start treatment .Time to end treatment .Therapy	T-stage .N-stage .M-stage .AJCC stage .Locoregional metastasis .Distant metastasis
Yu (2021)	USA	Roswell Park Comprehensive Cancer Center/ USA	Internal: 591 Train:70% Test:30%	NM	2-year overall	HNC	SCC	`Radiotherapy	.Age .Gender .BMI .Marital Status .Alcohol consumption .Smoking status .HPV .Hospitalized .Feeding tube type	.Treatment regimen .Radiotherapy delayed .Radiotherapy dose .Radiotherapy duration .Chemotherapy type	.Site .Clinical Stage .Pathological grading
Adeoye (2022)	Hong Kong	Hong Kong Hospital Authority Clinical Management System (HA-CMS) of the Queen Mary Hospital/Hong Kong	Internal: 313 Train: 80% Test: 20% External: 500 (synthetic)	Overall: 131 (42.7%) Disease-specific: 72 (24.1%)	Overall Disease-specific	Oral Cavity	SCC	Surgery (primary) Radiotherapy Chemotherapy	.Age .Gender .Previous cancer history	.Resection margin .Surgery plus radiotherapy or chemotherapy	.Tumor size .Nodal involvement .T-Stage .Tumor Grade .Histologic characteristics .Tumor site
Peng (2022)	China	National Cancer Institute (NCI) Surveillance, Epidemiology, and End Results (SEER)/ USA External: The Cancer Genome Atlas/USA	Interna: 64,226 Train: 90% Test: 10% External: 243	Overall: 54% Disease-specific: 39%	Overall Disease-specific	Oral Cavity Oropharynx Hypopharynx Larynx	SCC	Surgery Radiotherapy Chemotherapy	.Age .Sex .Race .Marital status .Insurance state .HPV status	.Surgery (yes/no) .Radiotherapy (yes/no) .Chemotherapy (yes/no)	.Tumor site .Year of diagnosis .Second cancer .Tumor number .Tumor size .Extension .Lymph metastasis .Histological type, .Grade, .TNM stage .T stage .N stage .M stage
Kim (2022)	South Korea	National Cancer Institute (NCI) Surveillance, Epidemiology, and End Results (SEER)/ USA	Internal: 4039 Train: 70% Test: 30%	Disease specific: 515 (12:7%) Non-specific: 779 (19.2%)	7-year overall 7-year disease-specific	Oropharynx	SCC	Surgery Radiotherapy Chemotherapy	.Age .Sex .HPV	.Surgery (yes/no) .Neck dissection (yes/no) .Radiotherapy (yes/no) .Chemotherapy (yes/no)	.Tumor grade .Tumor size .T stage .N stage .M stage .Clinical stage
Alabi (2022)	Finland	National Cancer Institute (NCI) Surveillance, Epidemiology, and End Results (SEER)/ USA	Internal: 3284 Train: 3164 Test: 120	Year of diagnosis 2010 (8.4%) 2011 (12.3%) 2012 (16.4%) 2013 (19.4%) 2014 (21.8%) 2022 (21.7%)	Overall	Oropharynx	SCC	Surgery Radiotherapy Chemotherapy	.Gender .Ethnicity .Marital status .Tumor grade .HPV status	.No treatment .Surgery (yes/no) .Surgery plus Chemotherpay .Definitive chemoradiotherapy .Surgery plus post radiotherapy	.T-stage .Nodal status .M-stage .Tumor site
Tan (2022)	Hong Kong	Queensland Cancer Registry (QCR)/Australia	Internal: 3841 Train: 70% Test: 30%	3-year: 40.9% 5-year: 51.0%	3-year overall 5-year overall	Oral cavity	SCC	NM	.Age .Sex .Local government Area	NM	.Tumor site .Tumor differentiation
Liao (2023)	China	National Cancer Institute (NCI) Surveillance, Epidemiology, and End Results (SEER)/ USA	Internal: 6316 Train: 4237 Test: 2079	NM	Overall	Larynx	SCC	Surgery Radiotherapy Chemotherapy	.Age .Sex .Race .Marital status	.Surgery (yes/no) .Radiotherapy (yes/no) .Chemotherapy (yes/no)	.T-stage .N-Stage .M-Satge .AJCC stage .Tumor size .Tumor differentiation .Tumor site
Alabi (2023)	Finland	National Cancer Institute (NCI) Surveillance, Epidemiology, and End Results (SEER)/ USA	Internal: 428 Train: 428	261 (61.4%)	5-year overall	Oral Cavity (Tongue)	SCC	Surgery	.Age .Gender .Ethnicity .Marital status	.Surgery only . Adjunctive radiotherapy (yes/no) . Adjunctive chemotherapy (yes/no) .None	.T-stage .N-stage .M-stage .Grade
Kotevski (2023a)	Australia	Prince of Wales Hospital Cancer Centre (POWCC) MOSAIQ oncology information system (OIS)/ Australia	Internal: 1105 Train: 80% test: 20%	15%	2-year disease-specific	HNC Oral Cavity Oropharynx Larynx	SCC	Radiotherapy	.Age .Sex .Hypothyroidism .Alcohol consumption .Fitness for operation .Performance status	.Surgery (yes/no) .RT dose .RT Fractions .RT duration .Chemotherapy (yes/no)	.Tumor site .Tumor grade .Cancer oeprable .T-satge .N-stage .TNM stage
Kotevski (2023b)	Australia	Oncology information systems (OIS): MOSAIQ and ARIA, from seven major tertiary referral hospitals in NSW, Australia,	Internal: 2953 Train: 80% Test: 20%	548 (18%)	2-year disease-specific	HNC Oral Cavity Oropharynx Larynx Nasopharynx Hypopharynx	SCC	Radiotherapy	.Age .Sex .Hospital location	.EQD2T .Surgery (yes/no_ .Chemotherapy (yes/no)	.Tumor site .T-stage .N-Stage
Sun (2023)	China	National Cancer Institute (NCI) Surveillance, Epidemiology, and End Results (SEER)/ USA	Internal: 8677 Train: 6073 (70%) Test: 2604 (30%)	NM	3-year Overall 5-year Overall	Larynx	SCC	Radiotherapy Surgery Chemotherapy	.Sex .Age .Race .Marital Status	.Surgical techniques (Endoscopic surgery Excisional biopsy Partial laryngectomy Total laryngectomy Removal of lymph node) .Radiotherapy (yes/no) .Chemotherapy (yes/no)	.Tumor site in larynx .Laterality of Tumor .Extent .Regional nodes being positive .AJCC grade .T stage .N- stage .Histologic differentiation grade
Choi (2023)	South Korea	NM/South Korea	Internal: 1026	154 (15.1%)	5-year Overall Recurrence-free Metastasis-free Local-Recurrence-Free Regional-Recurrence Free	Larynx	SCC	Radiotherapy Surgery	.Age .Sex, .Smoking .Alcohol consumption.ECOG	.Surgery .Surgery + adjuvant RT .Surgery + adjuvant CCRT .RT .CCRT	.Location of tumor .TNM stage .Recurrence
Li (2023a)	China	Beijing Tongren Hospital/China	Internal: 295 Train: 70% Test: 30%	178	3-year Overall	Hypopharynx	SCC	Surgery	.Age .Sex, .Smoking history .Drinking history	.Pre and post RT .Pre and post.Chemotherapy .Transoral laser microsurgery .Partial laryngectomy, .Partial hypopharyngeal resection .Total laryngectomy .Total hypopharyngeal resection .Skin flap repair	.Pathological differentiation .TNM stage .Clinical stage
Xiao (2023)	China	National Cancer Institute (NCI) Surveillance, Epidemiology, and End Results (SEER)/ USA	Internal: 1683 Train: 70% Test:30%	NM	Overall	Nasopharynx	SCC	Surgery Radiotherapy Chemotherapy	Age .Race .Sex .Marital Status .Ethnicity	.Surgery (yes/no) .Chemotherapy (yes/no) .Radiotherapy (yes/no) .Treatment regimen	AJCC stage .T-stage .N-stage .M-stage .Grade .Primary Site
Alabi (2024)	Finland	National Cancer Institute (NCI) Surveillance, Epidemiology, and End Results (SEER)/ USA	Internal: 2729 Train: 50% Test: 50%	1679 (60.1%)	Overall	Larynx	SCC	Surgery Radiotherapy Chemotherapy	.Age .Race .Gender .Marital Status	.Surgery (yes/no) .Chemotherapy (yes/no) .Radiotherapy (yes/no)	.AJCC stage .T-stage .N-stage .M-stage .Grade
Li (2024)	China	Zibo Central Hospital and Beijing Chaoyang Hospital /China	Internal: 150 Train: 60% Test: 40%	45	5-year Overall	Larynx	SCC	Radiotherapy Chemotherapy	.Age .Gender .Smoking Status .Alcohol consumption	Chemotherapy (yes/no) Radiotherapy (yes/no)	.AJCC stage .T-stage .N-stage .M-stage .clinical stage .Recurrence .Pathological Type

Abbreviations

NM: not mentioned, HNC: head and neck cancer, AJCC: American Joint Committee on Cancer, EQD2T: Equivalent Dose in 2 Gy fractions; CCRT: concurrent chemoradiotherapy; SCC: squamous cell carcinoma

**Table 3:** The characteristic of the included studies in chronological order for the disease progression outcomes.

First Author (Year)	Authors’ Affiliation	Data Source/ Population	Sample Size	Cases Frequency N (%)	Disease Progression Outcome Type	Tumor Site	Primary Treatment	Clinicopathological Features Included in Models		
								Patient-related	Treatment-related	Tumor-related
Tseng (2015)	Taiwan Republic of China	A Single Center Hospital/Southern Taiwan	Internal: 673 Train: 90% Test (10%)	Recurrence: 131	Recurrence	Oral cavity	Surgery	.Age .Sex .Oral history .smoking .Alcohol .Betel-nut chewing .Family history	.Duration between onset and the beginning of treatment .CCRT (yes/no)	.Tumor site .AJCC-N .Metastasis level in lymph node .Lymphovascular invasion .Peripheral nerve invasion .Tumor size .Clinical stage .Distant metastasis
Cheng (2018)	Taiwan	Cancer registries of three hospitals in Taiwan/Taiwan	Internal: 1428 Train: 90% Test (10%)	NM	Recurrence	Oral Cavity	Surgery Radiotherapy	.Age .Body mass index .Smoking .Betel nut chewing drinking	.Surgery (yes/no) .Surgical margin .Radiotherapy (yes/no) .Dose to clinical target volumes .Radiotherapy + Surgery .Sequence of local regional therapy and systemic therapy	.Tumor site .Histology .Differentiation .Behavior code .Tumor size .Pathologic stage
Alabi (2019)	Finland	University Hospitals of Helsinki, Oulu, Turku, Tampere, and Kuopio/Finland A.C. Camargo Cancer Center in Sao Paulo/Brazil	Internal: 311 Train: 70% Test: 30% External: 59	89 (28.6%)	Recurrence	Oral Cavity (Tongue)	Surgery	.Age . Gender	NM	.T stage .WHO histologic grade .Depth of invasion .Tumor budding .Worst pattern of invasion .Perineural invasion .Lymphocytic host response
Alabi (2020)	Finland	Patients treated at the five Finnish University Hospitals of Helsinki, Oulu, Turku, Tampere, and Kuopio and at the A.C. Camargo Cancer Center, Sao Paulo, Brazil/Finland and Brazil	Internal: 311 Train: 50% Test: 50% External: 59 (Brazil)	68 (26.8%)	Recurrence	Oral Cavity (Tongue)	Surgery (primary) Radiotherapy Chemotherapy	.Age .Gender	.Neck dissection (yes/no) .Surgery plus radio- or chemotherapy (yes/no)	.T-Stage .Grade .Tumor budding .Tumor Depth .Worst pattern of invasion .Lymphocytic host response .Perineural invasion
Chu (2020)	Hong Kong	Queen Mary Hospital in Hong Kong/ Hong Kong	Internal: 408 Train: 408	Recurrence: 131 (32%) Survival: 151 (37%)	Recurrence/metastasis	Oral Cavity	Surgery (primary) Radiotherapy Chemotherapy	.Age .Sex .Smoking history .Alcohol drinking .HPV status Categorical .EBV status .Past cancer history .Epstein-Barr virus .Human papillomavirus	.Neck dissection . Adjunctive Radiotherapy .Adjunctive Chemotherapy	.Second primary tumor .T-stage .N-stage .Disease Stage .Frozen section result .Resection margins result .Tumor grading .Depth of invasion .Extra-nodal extension .Lymphovascular invasion .Perineural invasion .Tumor site
Shan (2020)	China	Department of Oral and Maxillofacial Surgery, The Affiliated Stomatological Hospital of Nanjing Medical University/Chinna	Internal: 145 Train: 70% Test: 30%	56	Cervical lymph node metastasis	Oral Cavity (Tongue)	.Surgery	.Age .Gender .Tobacco .Alcohol use	NM	.Tumor Site .T- Stage .Morphology .Histological Grade .Depth of invasion .Neural infiltration .Muscle infiltration .Tumor budding .Nodal Status
Bourdillon (2022)	USA	Yale-New Haven Health system tumor registry/USA	Internal: 398 Train: 80% Test: 20%	102 (26.2%)	Recurrence across four one-year intervals	Oral cavity	Surgery (primary) Radiotherapy Chemotherapy	.Age .Sex .Race .Insurance status .Tobacco use .Alcohol use .Charlson-Deyo Comorbidity Index (CCI)	.Surgery (yes/no) .Time diagnosis to surgery .Surgical margin status .Neck dissection type .Lymph node yield .Number of positive lymph nodes .Adjunctive Radiotherapy .Adjunctive Chemotherapy	.Tumor site .T-stage .N-stage .AJCC best stage .Grade .Perineural invasion .Lymphovascular.invasion .Extranodal extension
Gangli (2022)	2022	One hospital/India	Internal: 311 Train: NM Test: NM	Locoregional recurrence: 54 Distance recurrence: 30	Locoregional recurrence Distance recurrence	HNC	Radiotherapy	. Presenting symptoms . Addictions/substance abuse . Comorbidities	. Primary treatment details (surgical, radiotherapy, and chemotherapy details) . Acute and late toxicities	. site of Cancer . The extent of primary and Lymph nodes . Clinical and pathological staging . Histopathological details
Cai (2023)	China	Sun Yatsen Memorial Hospital/China	Internal: 387 Train: 80% Test: 20%	52 (13.4%)	Recurrence (five-year)	Oral Cavity	Surgery	.Age .Sex .Hieght .Weight, Smoking .Drinking history.Systematic medical history .Previous treatment history	.Post-op radiotherapy .Post-op chemotherapy	.Tumor site .TNM staging .Timely and long.Operation time,.Postoperative complications .Postoperative pathological data .HPV status .Vitamin D level .Preoperative and postoperative blood result .Postoperative recurrence time .Postoperative death time
Zhang (2023a)	China	Eye & ENT Hospital of Fudan University	Internal: 671 Train: 70% Test: 30%	Progression-free: 73.7%	Recurrence/metastasis	Larynx	Surgery	.Age .Gender .Marriage .Smoking .Alcohol . Hypertension/diabetes	.Pre-operative chemo-radiotherapy .Surgery (including the types) . Neck lymph node dissection .Post-operative time . Postoperative chemoradiotherapy . Resection margins	.T stage .N stage .Clinical stage .Volume .Pathology Grading
Fataour (2023)	USA	National Cancer Institute (NCI) Surveillance, Epidemiology, and End Results (SEER)/ USA	Internal: 5-year:14,995 10-year: 7,342 Train: 80% Test: 20%	5-year: 657 10-year: 971	Recurrence (5-year) Recurrence (10-year)	Oral Cavity (Tongue)	Surgery Radiotherapy Chemotherapy	.Age .Sex .Race .Marital Status	.Surgery (yes/no) .Radiotherapy (yes/no) .Chemotherapy (yes/no)	.Number of Prior Tumors .Histology .Tumor Grade T-stage N-stage M_stage

Abbreviations

NM: not mentioned, AJCC: American Joint Committee on Cancer; CCRT: concurrent chemoradiotherapy; SCC: squamous cell carcinoma

According to Table 4, 11 out of 24 studies conducted time-to-event analyses for the survival outcome. In contrast, 14 studies performed binary classification, and one study carried out regression task. Also, six out of 24 studies performed calibration besides discrimination analyses. Referring to Table 5, for the disease progression outcome, only one study undertook time-to-event analyses, whereas the remaining 10 studies engaged in binary classification. Considering Tables 4 and 5 for both survival and disease progression outcomes, 12 studies implemented dimensionality reduction or feature selection, and also eight studies addressed the class imbalance through correction techniques. Concerning validation methods for the developed models, four studies utilized external validation, two applied temporal validation, and the remaining studies depended on internal validation.

**Table 4:** Performance of the included models in chronological order for the survival outcome.

First Author (Year)	Type of Analyses	Machine Learning Factors						Traditional Comparator/Performance Metrics
		ML Models	Dimensionality Reduction or Feature Selection	Resampling Techniques	Imbalanced Class Correction	Validation	Best Model/Performance Metrics	
Tseng (2015)	Binary Classification	1. ANNs 2. Decision Tree	None	Ten-fold cross-validation+Q4:S5	None	Internal	**Decision Tree/**Accuracy: 0.95	**Logistic Regression/**Accuracy: 0.56
Sharma (2015)	Binary Classification	1. Probabilistic neural network and general regression neural network (PNN,GRNN)	InfoGainAttributeEval by Rankler	Leave-one-out	None	Internal	**PNN, GRNN/** AUROC: 0.74 Accuracy: 0.69 Recall: 0.91 Specificity: 0.55 Precision: 0.58 F-1 score: 0.70	None
Karadaghy (2019)	Binary Classification	1. Random forest 2. Decision jungle 3. Logistic regression 4. Neural network	Permutation feature importance scores	None	None	Internal	**Random Forest/** AUROC: 0.80 Accuracy: 0.71 Precision: 0.71 Recall: 0.68 F1-score: 0.69 (manual)	**TNM/** AUROC: 0.68 Accuracy: 0.65 Precision: 0.69 Recall: 0.52 F1-score: 0.59 (manual)
Kim (2019)	Time-to-Event	1.DeepSurv 2. Random Survival Forest	None	hold out	None	Internal	**DeepSurv/** C-index: 0.78	**Cox PH/** C-index: 0.69
Hung (2020)	Regression	1. Decision tree 2. Random forest 3. XGBoost	Random Forest	Cross validation	None	Internal	**XGBoost/** MSE: 485.55 RMSE: 22.06 MAE: 13.55 R2 score: 0.711	**Linear Regression/** MSE: 647.49 RMSE: 25.45 MAE: 18.21 R2 score: 0.620
Alkhadar (2020)	Binary Classification	1. KNN 2. Naïve Bayes 3. Decision tree 4. Random forest	None	Five-fold cross-validation	Majority class under sampling	Internal	**Decision Tree/** AUROC: 0.77 Accuracy: 0.76 Recall: 0.81 Specificity: 0.72 Precision: 0.69 F1-score: 0.75	**Logistic Regression/** AUROC: 0.69 Accuracy: 0.60 Recall: 0.74 Specificity: 0.50 Precision: 0.53 F1-score: 0.62
Du (2020)	Time-to-Event Calibration	1. Survival Tree 2. Random Survival Forest 3. Conditional Inference Forest	None	Ten-fold cross-validation	None	Internal	**Random Survival Forest/** **3-year** C-index: 0.77 **5-year** C-index: 0.76	**Cox PH/** **3-year** survival C-index: 0.76 **5-year** survival C-index: 0.76
Nogay (2020)	Binary Classification	1. Nearest neighbor classifier 2. SVM 3. ANN 4. Decision Tree 5.Linear Discriminant	None	Hold out	None	Internal	**ANN/** AUCROC: 0.94 Accuracy: 0.90	**ANN/** AUCROC: 0.84 Accuracy: 0.86
Yu (2021)	Time-to-Event Binary classification	1. Random survival forest	None	Cross validation (k not mentioned)	None	Internal	**Random survival forest/** C-index: 0.70 AUROC: 0.75 Recall: 0.79 Precision:0.56 Specificity: 0.66 F1-score: 0.65	**Cox PH/** C-index: 0.63 AUROC: 0.71 Recall: 0.54 Precision:0.54 Specificity: 0.80 F1-score: 0.54
Adeoye (2022)	Time-to-Event Calibration	1. DeepSurv 2. DeepHit 3. Neural net-extended time-dependent cox model (Cox-Time) 4. Random Survival Forest	Cox PH	Five-fold cross-validation	None	External	**DeepSurv/** **Overall survival** C-index: 0.77 Integrated Brier scores: 0.20 **Disease-specific survival** C-index: 0.89 Integrated Brier scores: 0.13	None
Peng (2022)	Time-to-Event Calibration	1. Random Survival Forest 2. GBDT for survival 3. SVM for survival 4. ANN for survival 5. DL for survival	None	Ten-fold cross-validation	None	Internal External	**Random Survival Forest/** **Overall survival** **C-index for Sites:** Oral cavity: 0.80 Oropharyngeal: 0.79 Larynx: 0.77 Hypopharynx: 0.72 **Disease-specific survival** **C-index for Sites:** Oral cavity: 0.84 Oropharyngeal: 0.82 Larynx: 0.82 Hypopharynx: 0.79	**Cox PH/** **Overall survival** **C-index for subsites:** Oral cavity: 0.77 Oropharyngeal: 0.74 Larynx: 0.57 Hypopharynx: 0.55 **Disease-specific survival** **C-index for subsites:** Oral cavity: 0.82 Oropharyngeal: 0.78 Larynx: 0.79 Hypopharynx: 0.77
Kim (2022)	Time-to-Event Calibration	1. Extremely randomized survival tree (EST) 2. Conditional survival forest 3. DeepSurv	None	Ten-fold cross-validation	None	Internal	**DeepSurve/** C-index: 0.77 RSME (survival value): 39.74 RSME (predictive error): 4.03 MAE (survival value): 34.23 MAE (predictive error): 3.39 MAE: 0.10 IBS: 0.08	None
Alabi (2022)	Binary Classification	1. Voting ensemble 2. Light GBM 3. XGBoost 4. Random Forest 5. Extreme Random Trees	None	Five-fold cross-validation	None	Internal-External (temporal validation from the same dataset)	**Voting Ensemble/** AUROC: 0.93 Accuracy: 0.88 Mathews’ correlation: 0.72 Precision: 0.93 Recall: 0.91 Specificity: 0.82 F1-score: 0.92	None
Tan (2022)	Binary Classification	1. Random Forest 2. XGBoost 3. Gaussian Naïve Bayes 4. Voting Classifier	None	Five-fold cross-validation	SMOTE	Internal	**Voting Classifier/** **3-year** AUROC: 0.61 Accuracy: 0.59 Recall: 0.73 F1-score: 0.58 **5-year** AUROC: 0.63 Accuracy: 0.62 Recall:0.73 F1-score: 0.64	**Logistic Regression/** **3-year** AUROC: 0.61 Accuracy: 0.59 Recall: 0.73 F1-score: 0.57 **5-year** AUROC: 0.63 Accuracy: 0.62 Recall: 0.72 F1-score: 0.64
Liao (2023)	Time-to-Event	1. DeepSurv	None	Five-fold cross-validation	None	Internal-External (temporal validation from the same dataset	**DeepSurv/** C-index: 0.71	**TNM/** C-index: 0.61
Alabi (2023)	Binary Classification	1. K-nearest neighbor oracle (KNORA) 2. Extreme random trees	Cox PH	Ten-fold cross-validation	None	Internal	**Extreme Random Trees/** AUROC: 0.91 Accuracy: 0.86 Recall: 0.80 F1-score: 0.86	None
Kotevski (2023a)	Binary Classification	1. Logistic regression 2. Gradient boosted trees 3. Random forest 4. SVM 5. Neural network	None	Five-fold cross-validation	Oversampling using SMOTE	Internal	**Random Forest/** **HNC** AUROC: 0.97 F1-score: 0.89 **Larynx** AUROC: 0.97 F1-score: 0.92 **Oral Cavity** AUROC: 0.67 F1-score: 0.71 **Oropharynx** AUROC: 0.93 F1-score: 0.90	**Logistic Regression/** **HNC** AUROC: 0.76 F1-score: 0.77 **Larynx** AUROC: 0.92 F1-score: 0.92 **Oral Cavity** AUROC: 0.52 F1-score: 0.62 **Oropharynx** AUROC: 0.84 F1-score: 0.82
Kotevski (2023b)	Binary Classification Time-to-Event Calibration	1. Random Survival Forest 2. Gradient Boosted trees	None	Five-fold cross-validation	Oversampling using SMOTE	Internal	**Random Survival Forest/** **HNC** C-index: 0.79 AUROC: 0.93 F1-score: 0.88 **Oral Cavity** C-index: 0.73 AUROC: 0.85 F1-score: 0.79 **Larynx** C-index: 0.82 AUROC: 0.97 F1-score: 0.91 **Oropharynx** C-index: 0.79 AUROC: 0.95 F1-score: 0.85 **Hypopharynx** C-index: 0.73 AUROC: 0.85 F1-score: 0.77 **Nasopharynx** C-index: 0.83 AUROC: 0.95 F1-score: 0.90	**Cox PH/** C-index: 0.70
Sun (2023)	Time-to-Event Calibration	1. Random Survival Forest	LASSO	Ten-fold cross validation	None	Internal	**Random Survival Forest/** **3-year** C-index: 0.82 **5-year** C-index: 0.79	**Cox PH/** 3-year survival C-index: 0.72 5-year survival C-index: 0.72
Choi (2023)	Time-to-Event	1. DNN multi-classification 2. DNN regression 3. Random Survival Forest	None	Five-fold cross-validation	None	Internal	**DNN Classification/** Overall Survival C-index: 0.85	**Cox PH/** Overall Survival C-index: 0.74
Li (2023)	Binary Classification	1. Random Forest 2. Logistic regression 3. K-nearest neighbor 4. Decision Tree 5. AdaBoost 6. Multilayer perceptron 7. XGBoost 8. SVM 9. Linear discriminant analysis 10. Light GBM 11. CATBoost	None	None	None	Internal	**XGBoost/** AUROC: 0.77 Accuracy: 0.80 Sensitivity: 0.92 Specificity: 0.62	**Logistic Regression/** AUROC: 0.69 Accuracy: 0.73 Sensitivity: 0.87 Specificity: 0.51
Xiao (2023)	Time-to-Event	1. Random survival forest 2. Survival SVM	None	Hold out	None	Internal	**Survival SVM/** C-index: 0.78	**Cox PH/** C_index: 0.72
Alabi (2024)	Binary Classification	1. DeepTables 2. Voting Ensemble 3. Stack Ensemble 4. XGBoost	None	Five-fold cross-validation	None	Internal	**XGBoost/** AUROC: 0.76 Recall: 0.66 Precision: 0.60 F1-score: 0.63 Specificity:0.75 Accuracy: 0.71 Mathew’s correlation: 0.40	None
Li (2024)	Binary Classification	1. Random Forest 2. Logistic regression 3. K-nearest neighbor 4. Decision Tree 5. AdaBoost 6. Multilayer perceptron 7. XGBoost 8. SVM	None	None	None	Internal	**SVM/** AUROC: 0.81 Accuracy: 0.85 Sensitivity: 0.87 Specificity: 0.75	**Logistic Regression/** AUROC: 0.77 Accuracy: 0.81 Sensitivity: 0.85 Specificity: 0.66

Abbreviations

ANN: artificial neural network; KNN: k-nearest neighbors; SVM: support vector machine; DNN: deep neural network; XGBoost: eXtreme Gradient Boosting, TNM: tumor, node, metastasis; Cox PH: Cox Proportional Hazards; GBDT: gradient boosting decision tree; GBM: gradient boosting machine; DL: deep learning; SMOTE: synthetic minority oversampling technique; LASSO: least absolute shrinkage and selection operator

**Table 5:** Performance of the included models in chronological order for the disease progression outcomes.

First Author (Year)	Type of Analyses	Machine Learning Factors						Traditional Comparator/Performance Metrics
		ML Models	Dimensionality reduction or Feature Selection	Resampling Techniques	Imbalanced Class Correction	Validation	Best Model/Performance Metrics	
Tseng (2015)	Binary Classification	ANNs Decision Tree	None	Ten-fold cross-validation	None	Internal	**Decision Tree/** Accuracy: 0.81	**Logistic Regression/** Accuracy: 0.67
Cheng (2018)	Binary Classification	1. KNN 2. KSTAR (instance-based classifiers) 3. Randomizable Filtered Classifier (RFC) 4. Random Tree	GainRaito	Ten-fold cross-validation	None	Internal	**KSTAR (instance-based classifiers)/** Accuracy: 0.77 Recall: 0.77 Specificity: 0.75 Fallout: 0.36 F1 score: 0.81 Matthews correlation: 0.50	None
Alabi (2019)	Binary Classification	ANNs	None	hold out	None	External	**ANNs/** AUROC: 0.97 Accuracy: 0.88 Recall: 0.71 Specificity: 0.98 Precision: 0.97 Negative predictive value: 0.84 F1-score: 0.81 (manually calculated)	**Logistic regression/**Accuracy: 0.86
Alabi (2020)	Binary Classification	1. SVM 2. Naïve Bayes 3. Boosted Decision Tree 4. Decision Forest	Permutation Feature Importance	Five-fold cross-validation	SMOTE	External	**Boosted Decision Tree/**Sensitivity: 0.79 Specificity: 0.83 Precision: 0.76 F1-score: 0.78 Accuracy: 0.81 NPV: 0.89 LR+: 4.65 LR-: 0.25	**Depth of invasion (DOI)/** Accuracy: 0.63
Chu (2020)	Binary Classification	1. Linear regression 2. Decision tree 3. SVM 4. KNN	Principle Component Analysis Univariate analyses	15-fold cross-validation	None	Internal	**Decision Tree/** AUROC: 0.67 Accuracy: 0.70 Sensitivity: 0.41 Specificity: 0.84	**Linear Regression/** AUROC: 0.68 Accuracy: 0.67 Sensitivity: 0.35 Specificity: 0.83
Shan (2020)	Binary Classification	1. Logistic regression 2. Random forest 3. SVM 4. Naïve base	None	Stratified K Fold (K not mentioned)	None	Internal	**SVM/** AUROC: 0.95 Sensitivity: 1.0 Specificity: 0.87	**Depth of invasion (DOI)/** AUROC: 0.61 Sensitivity: 0.91 Specificity: 0.31 **Neutrophil-to-lymphocyte ratio (NLR)/** AUROC: 0.53 Sensitivity: 0.53 Specificity: 0.53 **Tumor buddings/** AUCROC: 0.83 Sensitivity: 0.80 Specificity: 0.87
Bourdillon (2022)	Multiclass classification	1. Logistic regression 2. Decision tree 3. SVM 4. Artificial neural network	Chi-Square Nomogram	Five-fold cross-validation	None	Internal	**Decision Tree/** Primary metric: MAE: 0.65 Secondary metrics: Accuracy: 0.80 Recall: 0.80 Precision: 0.74 F1-score: 0.76 (manual)	**Logistic Regression/** Primary metric: MAE: 0.70 Secondary metrics: Accuracy: 0.79 Recall: 0.63 Precision: 0.79 F1-score: 0.70 (manual)
Gangli (2022/0	Binary Classification	1. Random forest 2. XGboost 3. SVM	Boruta Sequential forward floating selection	Ten-fold cross validation	SMOTE ADASYN	Internal	**SVM/** **Locoregional** AUROC: 0.94 Accuracy: 0.77 F1-score: 0.68 **Distance** AUROC: 0.99 Accuracy: 0.95 F1-score: 0.91	None
Cai (2023)	Binary Classification	1. Random Forest 2. Logistic regression 3. K-nearest neighbor 4. Decision Tree 5. Multilayer perceptron 6. SVM 7. Decision Tree 8. Gaussian Naïve Bayes 9. Bernoulli Naïve Base 10. Gradient Boosting	Univariate analyses	None	adaptive synthetic sampling (ADASYN)	Internal	**Multilayer perceptron/** AUROC: 0.88 Accuracy: 0.83 Recall: 0.74 F1-score: 0.53	**Logistic Regression/** AUROC: 0.69 Accuracy: 0.70 Recall: 0.50 F1-score: 0.30
Zhang (2023)	Time-to-Event	1. Random Survival Forest	None	None	None	Internal	**Random Survival Forest** C-index: not reported AUROC 1-year: 0.73 AUROC 3-year: 0.64 AUROC 5-year: 0.64	**Cox PH** C-index: 0.60 AUROC 1-year: 0.67 AUROC 3-year: 0.71 AUROC 5-year: 0.71
Fataour (2023)	Binary Classification	1. GBM 2. GLM 3. DRF 4. Deep Learning		Five-fold cross-validation	Over sampling Under sampling	Internal	**GBM** **5-year recurrence:** AUROC: 0.75 Accuracy: 0.81 Recall: 0.83 Precision: 0.97 F1-score: 0.89 (manual) **10-year recurrence:** AUROC: 0.74 Accuracy: 0.80 Recall: 0.82 Precision: 0.94 F1-score: 0.87 (manual)	**GLM** 5-year recurrence: AUROC: 0.73 Accuracy: 0.77 Recall: 0.78 Precision: 0.98 F1-score: 0.86 (manual) 10-year recurrence: AUROC: 0.73 Accuracy: 0.78 Recall: 0.81 Precision: 0.87 F1-score: 0.87 (manual)

Abbreviations

ANN: artificial neural network, KNN: k-nearest neighbors, SVM: support vector machine, GBM: gradient boosting machine, GLM: generalized linear model, DRF: distributed random forest; MAE: mean absolute error; SMOTE: Synthetic minority oversampling technique

### 3.4. Performance of models for survival outcome

#### 3.4.1. Time-to-event models

For HNC as a whole, three studies reported C-indices ranging from 0.70 to 0.79 for ML models; In the same studies, the C-indices for Cox PH studies ranged from 0.66 to 0.76 [66, 68, 75]. For the oral cavity, the C-index for four studies conducting time-to-event analyses ranged from 0.73 to 0.89, with three of these studies also reporting on Cox PH models, showing C-indices between 0.69 and 0.77 [60, 71, 72, 75]. For the larynx, five studies reported C-indices for ML models ranging from 0.71 to 0.85, and for Cox PH models in the same studies, ranging from 0.57 to 0.74 [72, 75, 78, 80, 86]. For the oropharynx, three studies reported C-indices ranging from 0.77 to 0.80, [72, 73, 75], but there was not enough data to consolidate the C-index for Cox PH models. For the hypopharynx and nasopharynx, only two studies reported C-indices for ML models, ranging from 0.72 to 0.79 [72, 75] and from 0.72 to 0.83 [76, 77]. The C-index for Cox PH could not be consolidated for hypopharynx and nasopharynx.

Among the ML models, the Random Survival Forest (RSF) was employed in nine studies and compared with other ML models in seven of these studies. RSF outperformed the other models in three of these comparative studies [66, 72, 75]. Among the other four studies, two incorporated DeepSurv and demonstrated superior performance compared to RSF [60, 71]. Additionally, in another study, a deep neural network model [80] and survival SVM [77] outperformed RSF. Furthermore, DeepSurv excelled in all three studies in which it was compared to other models. The detailed performance metrics for the best-performing models can be found in Table 4.

#### 3.4.2. Binary classification models

For HNC as a whole, four studies reported AUROC ranging from 0.75 to 0.97, and three of these studies also reported F1-scores ranging from 0.65 to 0.89; based on three of these studies, AUROC for logistic regressions ranged from 0.71 to 0.84, and based on two studies, F1-scores ranged from 0.54 to 0.77 [67, 68, 75, 76]. For the oral cavity, seven studies reported AUROC and F1-score for ML models, ranging from 0.61 to 0.91 and 0.58 to 0.86, respectively, with four of these studies also reporting on logistic regression models, which ranged from 0.52 to 0.69 for AUROC and 0.57 to 0.62 for F1-score [56, 58, 63, 75, 76, 87, 88]. For the larynx, four studies reported AUROC for ML models ranging from 0.76 to 0.97, and three of these studies also reported F1-scores ranging from 0.63 to 0.92; from these four, two studies also reported on logistic regression with AUROC ranging from 0.76 to 0.92 [75, 76, 81, 85]. For the oropharynx, three studies reported AUROC for ML models ranging from 0.93 to 0.97 and F1-scores ranging from 0.90 to 0.92, but there was not enough information to consolidate results for logistic regression [74–76]. Moreover, the AUROC of ML models ranged from 0.77 to 0.85 for two studies that reported on the hypopharynx [75, 82]. The results for the nasopharynx could not be synthesized since only one study reported on the related metrics.

Among ML models, tree-based models were used more often and generally demonstrated superior performance in terms of AUROC and F1-scores compared to other algorithms. In nine studies that conducted comparative analyses, six found that tree-based models, including random forest and XGBoost, outperformed others [58, 63, 76, 82, 85, 87]. However, in one study, a voting classifier of random forest, logistic regression, and Gaussian Naïve Bayes was superior [88]. In another study, a Support Vector Machine (SVM) excelled [81], and in one study, neural networks showed superior performance [67]. The detailed performance metrics for the best-performing models can be found in Table 4.

### 3.5. Performance of models for disease progression outcomes

#### 3.5.1. Time-to-event models

The only study that performed time-to-event analyses did not report a c-index for the RSF model, but it was 0.60 for Cox PH [83].

#### 3.5.2. Binary classification models

Overall, out of 11 studies, nine studies reported on the performance of ML models on the oral cavity from which five studies reported AUROC values ranging from 0.67 to 0.97 [59, 64, 65, 79, 84]. Among these, three studies also evaluated traditional linear models, with the AUROC for corresponding ML models ranging from 0.67 to 0.88, while for linear models including logistic regression, it ranged from 0.68 to 0.73 [64, 79, 84]. Additionally, six studies provided F-1 scores for ML models, with values ranging from 0.53 to 0.89 [57, 59, 62, 69, 79, 84]. Of these, three studies included F1-scores for linear models as well, with the corresponding ML models’ F1-scores ranging from 0.53 to 0.89 and linear models’ scores from 0.30 to 0.87 [69, 79, 84]. Besides the oral cavity, other sites either had no or only one related study, so their results could not be consolidated.

For the disease progression outcomes, unlike the survival outcomes, there was not a consistent trend indicating superior performance of tree-based models. The specific performance metrics for the top-performing models are detailed in Table 5.

## 4. Discussion

This systematic review assessed the utilization and performance of ML models in predicting post-treatment survival and disease progression in HNC patients, based on structured clinicopathological data. ML models consistently surpassed traditional models, including Cox PH and logistic regressions, as well as nomograms derived from these linear models. Furthermore, the results indicated that, in time-to-event analyses, ML models demonstrated superior performance for specific HNC subsites, such as the oral cavity, oropharynx, and larynx, compared to overall HNC. This discrepancy may be attributed to the distinct risk factors, etiology, tumor biology, incidence, treatment strategies, and prognosis associated with each subsite. [89].

The enhanced performance of ML models in modeling time and censored data for the survival outcome of different sites, including HNC as a whole (C-index range: 0.70 to 0.79), oral cavity (C-index range: 0.73 to 0.89), and larynx (C-index range: 0.71 to 0.85), compared to traditional models such as Cox PH for HNC (C-index range: 0.66 to 0.76), oral cavity (C-index range: 0.69 to 0.77), and larynx (C-index range: 0.57 to 0.74), can be attributed to several factors. Primarily, ML models are non-parametric, meaning they do not presuppose a specific form for the data and can also identify complex non-linear relationships between variables, which is beyond the capability of traditional linear models. Additionally, models such as Cox PH presume that the risks associated with different individuals are proportional over time, suggesting that at any given time point, a specific individual will always have a higher or lower risk than another, implying that their survival curves will never intersect, a premise not always valid in real-world settings. Moreover, multicollinearity is not an issue for most ML models, and they can process numerous features and their interactions, providing them with an intrinsic ability to dissect population-level incidence more effectively by considering a broader range of individuals’ features, thereby offering superior personalized risk predictions.

A variety of ML models were utilized for time-to-event analyses of the survival outcome. Among these models, DeepSurv emerged as the top performer. DeepSurv, a deep feed-forward neural network, outperformed the other models in every study in which it was employed [60, 71, 73]. DeepSurv implements a deep learning generalization of the Cox proportional hazards model and has an advantage over traditional Cox PH because it does not require a priori selection of covariates, but rather learns them adaptively. It has the capacity to model complex non-linear relationships between a patient’s covariates and the hazard function [90]. In the absence of DeepSurv, RSF showed the best performance in three studies [66, 72, 75]. RSF ensemble approach, combining multiple decision trees, enhances predictive accuracy, while its provision of variable importance measures offers valuable insights into influential factors affecting outcomes. RSF’s minimal preprocessing requirement is particularly beneficial in the medical field. Moreover, it adeptly handles non-linear relationships. Importantly, RSF allows for individual hazard functions to intersect, accommodating non-proportional hazards, which contrasts with models like DeepSurv that assume proportional hazards across individuals [91].

In the binary classification of survival outcomes, the observed trends paralleled those in time-to-event analyses with ML models outperforming linear models such as logistic regression. A broad spectrum of ML models was utilized for binary classification, encompassing simple neural networks, deep learning, SVMs, Naïve Bayes, K-nearest neighbors, gradient boosting, and various tree-based models. Among these, tree-based models, particularly random forest and XGBoost, were found to outperform other models in six out of nine studies that conducted such comparisons [58, 63, 76, 82, 85, 87]. However, there were instances where other algorithms were superior to tree-based models. For example, in one study, an SVM—distinguished by its capacity to identify the optimal margin separating different classes, particularly in high-dimensional spaces—exhibited superior performance [81]. Additionally, in another study, an ensemble voting model comprising random forest, logistic regression, and Gaussian Naïve Bayes excelled. This model considerably enhanced predictive accuracy by amalgamating the strengths of multiple models to reduce individual errors and variances [74]. This indicates that while tree-based models are favorable, there is a necessity to explore the potential of other ML models further.

In the examined research, two studies developed predictive models for both overall survival and disease-specific survival [71, 72]. Peng et al. observed that for the oral cavity, the performance of the RSF model was superior for disease-specific compared to overall survival based on the C-index score (0.84 vs 0.80). Another study by Adeoye et al. identified a consistent pattern showing that for the oral cavity, the C-index for disease-specific was higher than overall survival (0.89 vs. 0.77). The possible explanation for these findings can be that disease-specific survival only considers deaths attributed to the disease being studied, thus providing a more focused measure of a treatment’s effectiveness against the disease. In contrast, overall survival includes all causes of death, which can introduce more variability and potential confounding factors that could affect the model’s performance. Additionally, based on Table 2 and the chosen features, these results might be due to selecting predictors that are more tailored to the specific disease survival rather than overall survival.

The performance of the predictive models exhibited minimal variations across different follow-up durations. In the analysis of three studies comparing three- and five-year survival rates, two studies reported a marginally higher C-index for three-year survival (0.77 vs 0.76 and 0.82 vs 0.79) [66, 78], while another study, which utilized AUROC as a metric, found that the performance of ML models was negligibly higher for five-year vs three-year survival (0.63 vs. 0.61) [88]. These observed discrepancies may be related to the evolving nature of the diseases under study and the application of static variables at baseline in the models. While certain variables, such as patient socioeconomic status and health behaviors, may alter over time, the slight change in model performance suggests that baseline variables maintain consistent predictive value for extended follow-up periods compared to shorter ones.

In the analysis of disease progression outcomes among the 11 studies reviewed, none of the studies provided data on time-to-event metrics. However, according to binary classification metrics, ML models demonstrated superior performance compared to traditional linear regression models. The oral cavity was the only site with enough articles to synthesize the results. For ML models, the AUROC ranged from 0.67 to 0.88, while for linear models such as logistic regression models, the AUROC ranged from 0.68 to 0.73 for the same studies [64, 79, 84]. In the context of disease progression, in contrast to the findings on survival outcomes, there was no consistent pattern favoring a particular model, such as tree-based models. Given the limited number of studies and the scarcity of research on certain outcomes, further investigation is essential before drawing definitive conclusions.

The analyzed studies encountered several limitations, notably in the lack of comprehensive details on the treatments provided, such as surgical methods and dose-volume histogram information, which can impact both the development and performance of models. Additionally, while the majority of the research focused on survival outcomes, particularly overall survival, less attention was given to disease-specific outcomes and aspects of disease progression such as recurrences and metastasis, which significantly influence patient prognosis. Despite the emphasis on adhering to Transparent Reporting of a multivariable prediction model for Individual Prognosis (TRIPOD) reporting guidelines [92], only 11 out of 34 studies conducted time-to-event analyses and took into account the censored data. Moreover, the evaluation methods for the models predominantly focused on discrimination metrics, with only six studies including calibration to assess the alignment between predicted probabilities and actual outcomes, an essential component in model validation. Given the identified limitations, future research should focus on establishing more robust and transparent methodologies for ML modeling in HNC prognosis. It is imperative to conduct external validations to establish the generalizability of the models, which were notably scarce in the reviewed studies, with only four out of 34 studies undertaking this crucial step. Emphasizing the use of multicenter databases is also recommended to mitigate potential regional and demographic biases. Additionally, while ML models have shown promising results in HNC prognosis, there is a significant need for clinicians to be thoroughly educated on the nuances of each model, including their strengths, limitations, and biases. Such knowledge is crucial for clinicians to effectively leverage these models in clinical settings, promoting their broader adoption and integration into clinical practice.

## 5. Conclusion

ML models exhibit considerable potential in predicting post-treatment survival and progression in HNC patients. ML models consistently outperformed traditional linear models, such as logistic regression and Cox PH, as well as the nomograms derived from these models. Among ML models, DeepSurv followed by tree-based models demonstrated the highest performance. Regarding survival outcomes, models focusing on disease-specific outcomes achieved higher performance compared to those targeting overall survival, while there was no meaningful difference between follow-up durations of three and five years. There were fewer models for disease progression outcomes, with only one conducting time-to-event analyses. The studies generally lacked detailed incorporation of treatment specifics into their models, which could potentially improve model performance. Future research should integrate more comprehensive treatment data, place a greater emphasis on disease progression outcomes, and establish model generalizability through external validations and the utilization of multicenter datasets.

## Supporting information

S1 Checklist PRISMA 2020 checklist. (DOCX)
